# Integration and regulation of stolen organelles in a marine protist

**DOI:** 10.1093/ismejo/wrag204

**Published:** 2026-08-04

**Authors:** Julian M Jacobs, Zachary L Reitz, Erica Lasek-Nesselquist, Amelie L'Etoile-Goga, Brittany N Sprecher, Matthew D Johnson, Holly V Moeller

**Affiliations:** Department of Ecology, Evolution, and Marine Biology, University of California, Santa Barbara, Santa Barbara, CA 93106, United States; Biology Department, Woods Hole Oceanographic Institution, Woods Hole, MA 02543, United States; Department of Ecology, Evolution, and Marine Biology, University of California, Santa Barbara, Santa Barbara, CA 93106, United States; Wadsworth Center, New York State Department of Health, Albany, NY 12237, United States; Department of Biomedical Sciences, State University of New York at Albany School of Public Health, Rensselaer, NY 12222, United States; Department of Ecology, Evolution, and Marine Biology, University of California, Santa Barbara, Santa Barbara, CA 93106, United States; Biology Department, Woods Hole Oceanographic Institution, Woods Hole, MA 02543, United States; Biology Department, Woods Hole Oceanographic Institution, Woods Hole, MA 02543, United States; Department of Ecology, Evolution, and Marine Biology, University of California, Santa Barbara, Santa Barbara, CA 93106, United States

**Keywords:** Mesodinium, kleptoplasty, mixotrophy, kleptokaryon, sequestered prey organelles, plastid retention, host-organelle integration, acquired metabolism, acquired phototrophy

## Abstract

Chloroplast-stealing (a.k.a., “kleptoplastidic”) plankton transiently obtain and integrate prey metabolic machinery into their own cells. To do so, they must address a series of challenges, including physical localization of acquired machinery, metabolic integration of organelles and their products, and maintenance of machinery. Here, we study this transient integration in the marine ciliate *Mesodinium chamaeleon* by pulse feeding the ciliate with cryptophyte algal prey (*Storeatula major*) and then tracking changes in ciliate cellular ultrastructure, physiology (growth and photosynthesis), and gene expression over time. We demonstrate a predictable series of changes, from the reconfiguration of metabolic activity fueled by recent ingestion, to a period of rapid growth, to a reduction in physiological performance as prey organelle number and functionality become limiting. Because *M. chamaeleon* also steals transcriptionally active prey nuclei, gene expression is a complex milieu of host and cryptophyte expression, with much of prey metabolism apparently intact in the new host, albeit at low levels. Collectively, our results highlight the holistic orchestration of stolen organelles to produce rapid—yet transient—growth in a new host.

## Graphical Abstract

**Graphical Abstract:**
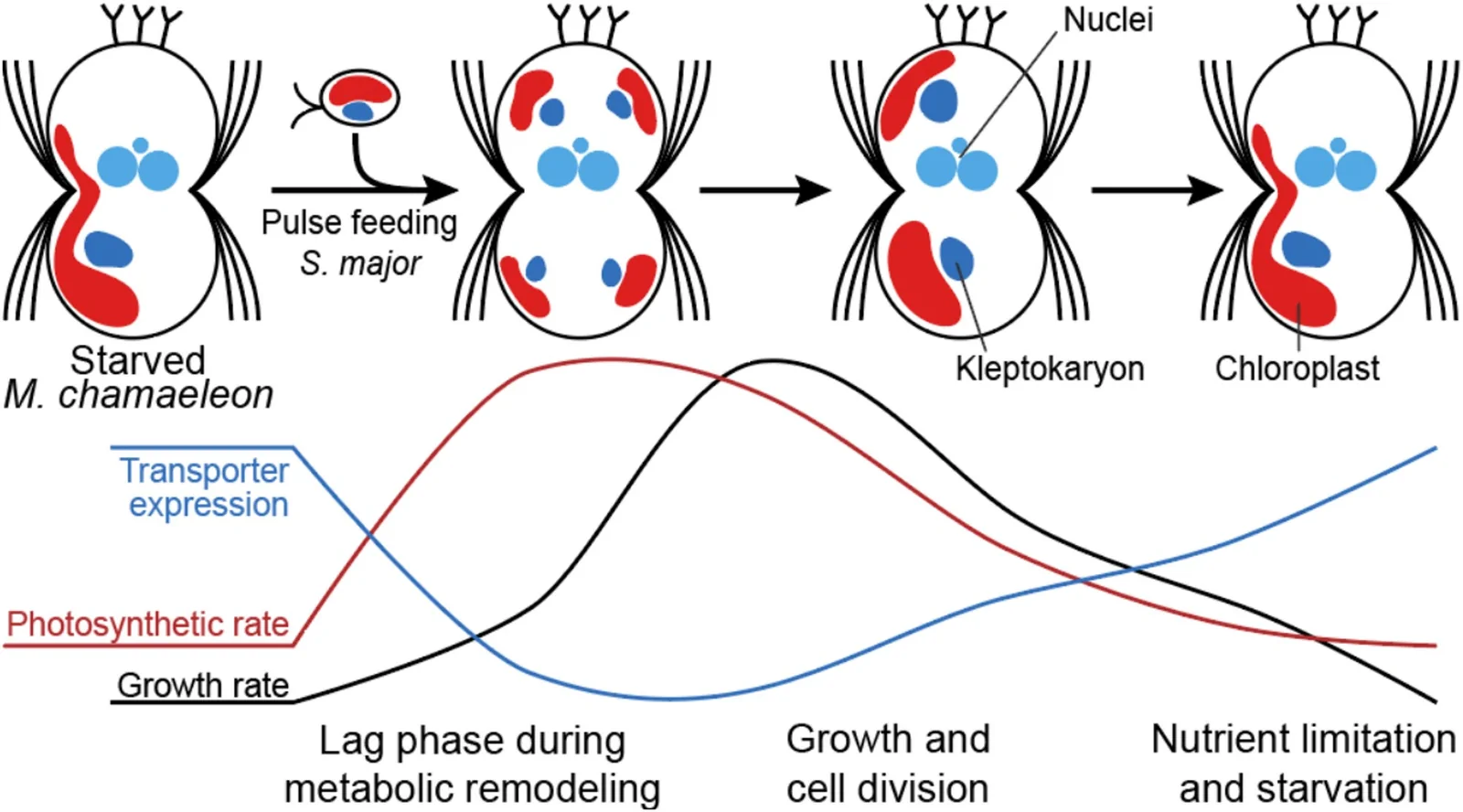

## Introduction

Kleptoplasty is the process by which an organism transiently harbors and metabolically incorporates a functional chloroplast from an ingested cell. Kleptoplasty occurs in a wide range of phagotrophic protist groups including foraminifera, dinoflagellates, euglenozoans, centrohelids, and ciliates [1–5]. These acquired phototrophs may benefit from products derived from harbored plastids, other sequestered prey organelles, and dual modes of metabolism. For example, photosynthetically active plastids can serve as a significant source of organic carbon and energy for a host [5, 6], and a mixture of phototrophic and heterotrophic metabolism (mixotrophy) provides physiological flexibility that can be advantageous in environments that are dynamic in growth-limiting factors such as prey, light, and nutrients [6].

Kleptoplastidic species are able to take advantage of the photosynthetic and anabolic potential of sequestered prey without the genetic and regulatory burden of vertically integrating additional genes and endogenously maintaining acquired organelles [7, 8]. However, these genetically heterotrophic organisms must continually capture prey and obtain new organelles to maintain photosynthetic output [1, 9]. Furthermore, hosts must delay prey digestion, handle destructive photochemical oxidative stress, and configure their metabolism to take advantage of obtained metabolites [10]. This has resulted in diverse prey retention strategies spanning a spectrum of prey retention duration, degree of prey digestion, and integration of prey organelles [4, 6]. Investigating the remodeling that coincides with the retention of photosynthetic organelles can provide insights into the cellular and physiological tradeoffs of kleptoplasty and identify the mechanisms that modulate cellular integration and complex plastid acquisitions [5, 7, 11].

The *Mesodinium* genus of ecologically important and cosmopolitan marine ciliates is a model system for studying kleptoplasty. This genus contains extant planktonic and benthic species that vary in their degree of plastid reliance and integration without any known nuclear-encoded genes to regulate the process [1, 12, 13]. The *Mesodinium chamaeleon/M. coatsi* species complex is a physiological intermediate between the heterotrophic *M. pulex/M. pupula* species complex and highly photosynthetic *M. rubrum/M. major* species complex [14] in terms of prey specialization [15, 16], prey organelle handling [15, 17], and metabolism [17, 18]. The existing ecophysiological and molecular data on the genus make *M. chamaeleon* a powerful candidate to study the cellular and physiological rewiring associated with prey consumption in kleptoplastidic lineages [7].

*Mesodinium chamaeleon* acquires about half of its carbon budget from functioning plastids and half from digestive heterotrophic activity [11, 14]. The ciliate can retain organelles from at least six cryptophyte species, including those with blue-green and red plastids [15, 16, 19]. Upon ingestion, prey organelles are housed in vacuoles around the periphery of the cell where the cryptophyte nucleus, or kleptokaryon (KN), also remains transcriptionally active [20]. However, kleptokarya within *M. chamaeleon* do not seem to facilitate the same plastid longevity or plastid division as in *M. rubrum* [16, 18] which have the capacity to maintain and divide numerous kleptoplastids via a single transcriptionally active kleptokaryon [21]. Ultimately, in *M. chamaeleon*, prey items are digested, expelled, or diluted across generations if not replenished by feeding, and high growth rates appear to be contingent on continuous replacement of sequestered plastids [15, 16, 20]. Although kleptoplasty has been proposed as an intermediate stage to vertically integrating plastids through organellogenesis by facilitating gene transfer in euglenozoans [3] and dinoflagellates [10], modeling the adaptive dynamics of *M. chamaeleon* has also revealed that kleptoplasty is a potentially evolutionary stable state when strong tradeoffs arise between chloroplast retention and digestive ability under certain environmental conditions [22].

In this study, we used *M. chamaeleon* to understand the heretofore poorly understood molecular and cellular mechanisms that underlie kleptoplasty [5]. Specifically, we quantified physiology and gene expression in *M. chamaeleon* and its sequestered cryptophyte organelles during starvation and at 1, 2, 4, and 7 days after a single pulse feeding event, capturing the initiation, maximum functioning, and degradation of a kleptoplastidic relationship. We hypothesize that high prey turnover is caused by decline in the functionality of sequestered organelles which results in the loss of photosynthetic and anabolic capacity over time. We found evidence of two distinct growth limitation phases: (i) an initial delay that arises from host cellular remodeling upon pulse feeding and (ii) terminal carbon limitation due to reduced photosynthetic capacity and nitrogen limitation during starvation. Our exploration of molecular dynamics in *M. chamaeleon* prey processing is an important first step in understanding the mechanisms that support the integration of photosynthesis and prey organelles in the species.

## Materials and methods

To quantify the effects of feeding history on *M. chamaeleon*, we conducted a pulsed feeding experiment in which *M. chamaeleon* cells were first offered their preferred prey, the cryptophyte *Storeatula major* [18–20], and then starved and interrogated with physiological and transcriptomic assays over time.

### Experimental design

We used *M. chamaeleon* culture NRMC-1802 [16], maintained in batch culture in sterile coastal filtered seawater at a temperature of 18°C with a photoperiod of 12-h light (at a light level of 10 μmol quanta m^−2^ s^−1^): 12-h dark. During normal culture maintenance, *M. chamaeleon* was fed its preferred prey, *S. major* strain “g” [18], once weekly at an ~10:1 ratio of prey to *M. chamaeleon. S. major* was maintained in sterilized coastal filtered seawater amended with f/2-Si nutrients [23], to reflect coastal marine conditions in which natural populations are found.

To poise *M. chamaeleon* cells with low levels of prey organelles, we starved *M. chamaeleon* cells for 21 days, monitoring population size every 1–2 days for the final 14 days of the starvation period (Fig. S1, Top). On the 21st day of starvation (Experimental Day 0), we pulse-fed *M. chamaeleon* at a 10:1 ratio of *S. major* prey cells to *M. chamaeleon* cells. By feeding at this ratio, we ensured that the majority of prey uptake occurred within the first 48 h of prey introduction. Indeed, the ratio of *M. chamaeleon* to *S. major* cells decreased exponentially after feeding until prey was completely cleared on Day 4 (Fig. S2, Bottom). For the following 7 days, we monitored the cultures through initial feeding to the onset of starvation (prey:predator ratios <0.1), with daily cell counts, and photophysiology and transcriptomic data collection on Experimental Day 0 (= “Starved” treatment), and at 1, 2, 4, and 7 days post-feeding. The experiment was run with six replicates with an initial 400 ml volume in 500 ml-capacity suspension cell culture flasks (CellTreat Scientific Products).

### Expansion microscopy & confocal laser scanning microscopy

We performed ultrastructure expansion microscopy [24, 25] to obtain high-resolution images of prey organelle sequestration dynamics over the experimental timeline. Samples for expansion microscopy originated from a secondary experiment identical to the one described above with the addition of one timepoint at 5 h post-feeding. For imaging, polyacrylamide gels embedded with cells were stained with the nucleic acid stain SYBR Green (Thermo Fisher Scientific) and labeled with anti-photosystem II D1 protein (PhytoAB), anti-Centrin (Millipore), and anti-α,ꞵ tubulin (ABCD Antibodies) using immunohistochemistry techniques to observe the cryptophyte periplast structure, photosystems, and cytoskeletal structures, respectively.

Gels were labeled with primary antibodies at a final concentration of 0.004 μg μl^−1^ in 4% bovine serum albumin (BSA) and 0.1% Tween 20 in phosphate-buffered saline (PBS-T) for 12 h at room temperature under gentle agitation (rotation). SYBR Green was included in the primary antibody solution to a final concentration of 2 μM. Gels were thoroughly rinsed with PBS-T to remove unattached labels and stain, and then, secondary antibodies were introduced in 4% BSA and 0.1% PBS-T to a final concentration of 0.002 μg μl^−1^ and rotated overnight at room temperature. The selected primary and secondary antibodies were cultivated in distinct hosts to ensure target-specific labeling. All samples were rinsed with PBS-T and then expanded in ultrapure water before mounting and imaging. Three-dimensional z-stacks were obtained on a Leica SP-8 Scanning Resonant Confocal Microscope. Each channel was then compressed into the maximum-intensity projection and layered as a composite using FIJI from ImageJ.

### Population dynamics

To monitor the population sizes and growth rates of *M. chamaeleon* cells, we fixed a 1 ml subsample of each culture daily using acid Lugol’s Solution at a final concentration of 1%. We then determined the abundances of *M. chamaeleon* and *S. major* (where present) by counting cells on a gridded Sedgwick Rafter counting chamber and computed the concentration of each population in cells ml^−1^. We determined growth rates by fitting a linear model to the natural log of population sizes as a function of experimental day, using a 3-day window of contiguous timepoints (e.g., the growth rate on Experimental Day 1 was based on population sizes from Days 0, 1, and 2). For the growth rate of Starved cultures (Day 0) and our final timepoint (Day 7), we used 3-day windows from Days −2 to 0 and Days 5–7, respectively, because population dynamics yielded consistent growth rates up to those timepoints (Fig. S2, middle).

### Photophysiology

To track photosynthetic machinery abundance and performance in *M. chamaeleon* cells following pulse-feeding, we measured chlorophyll content and electron transport (a proxy for photosynthetic rate) in *M. chamaeleon* cells. To do so, we first washed a 50 ml subsample of each experimental culture free of prey using an 8 μm Transwell membrane filter (Corning Scientific) [26]. This method allowed us to physically separate cryptophyte prey, which passed through the filter, from *M. chamaeleon* cells, which are retained on the filter [18]. For consistency in ciliate handling, we performed this washing for every photophysiology measurement regardless of the abundance of prey. After washing, we resuspended the prey-free *M. chamaeleon* in 50 ml of filtered seawater. We fixed a 1 ml subsample using acid Lugol’s Solution for enumeration to ensure accurate estimations of cellular chlorophyll content, and used a separate 5 ml sample for photophysiology measurements. The remainder of the washed sample was filtered onto 5 μm polytetrafluoroethylene (PTFE) membrane filters (Omnipore Membrane Filter Part No. JMWP04700, EMD Millipore) and flash-frozen in liquid N_2_ for later RNA extraction. All physiological and RNA sampling was done at 3 h into the 12-h light period to control for the effects of photoperiod.

We first used a fluorescence induction and relaxation (FIRe) system to quantify electron transport through photosystem II [26]. We specifically measured dark-acclimated photosynthetic efficiency (Fv/Fm) on cultures that had been dark-acclimated for at least 30 min prior to measurement and used an actinic light source to generate a photosynthesis–irradiance curve (PI curve) from 0 to 200 μmol quanta m^−2^ s^−1^ across 20 steps (0, 5, 10, 15, 20, 31.8, 43.5, 55.3, 67.1, 78.8, 90.6, 102.3, 114.1, 125.9, 137.6, 149.4, 161.2, 172.9, 184.7, and 196.5 μmol quanta m^−2^ s^−1^). Cells were acclimated to each increasing light level for 15 s before PSII was subjected to multiple turnover saturations achieved with a 100 ms flash, and fluorescence responses were measured. We fit the equation [27] to the PI curve data to obtain estimates of α at incident irradiance (*I*), the initial slope of the PI curve at low light levels, and *P*_max,chl_, the maximum per-chlorophyll photosynthetic rate from electron transport rates (ETRs):


$$ETR= Pmax\bullet \mathit{\tanh}\ \left(\frac{a\cdotp I}{Pmax}\right)$$

From this equation, we also calculated *P*_I,chl_, the per-chlorophyll photosynthetic rate at the growth irradiance of 10 μmol quanta m^−2^ s^−1^. We converted this value to the per-cell photosynthetic rate by using cellular chlorophyll content (see below).

Next, we filtered the same 5 ml sample onto a GF/F filter (Whatman) and placed that sample into 5 ml of 90% acetone for chlorophyll extraction. After an overnight (at least 20 h) incubation at −20°C, chlorophyll concentrations were quantified on a spectrophotometer (Turner Designs Trilogy). We used cell density of the washed *M. chamaeleon* samples to calculate cellular chlorophyll content.

### RNA sampling, extraction, and sequencing

We previously published the transcriptome data used in this study as part of a comparative analysis of *Mesodinium* spp. and provide full details on RNA-Seq data acquisition and processing therein [28]. Briefly, we extracted and sequenced RNA from three replicates for each *M. chamaeleon* treatment, as well as from free-living *S. major* prey using the RNeasy Plant kit (Qiagen) according to the manufacturer’s protocol. Illumina sequencing was performed at the DOE Joint Genome Institute Sequencing Center. We assembled transcriptomes *de novo* using megahit v.1.2.9 [29] and predicted proteins with Transdecoder v.5.7.1 (Haas, BJ) with the Mesodinium genetic code (#29 in NCBI’s “The Genetic Codes”) for *M. chamaeleon* samples. GhostKOALA [30], functionally annotated proteins by assigning them KO (KEGG Orthology) numbers. *Mesodinium chamaeleon* samples were extensively filtered at read, transcript, and protein levels to remove sequences from the kleptokaryon and contaminating bacteria according to methods described in a companion manuscript [28]. We analyzed transcription at the KO level by selecting one transcript to represent each KO for the macronucleus, kleptokaryon, and free-living prey (1694, 4343, and 4039 KOs, respectively). We then mapped filtered reads to the representative sequences and calculated per-KO counts using Salmon v.1.10.3 [31].

All downstream analyses were performed in R v.4.5.1. We considered KOs to be expressed if they consistently had 10+ counts across all replicates in at least one condition, and all other KOs were removed. Counts were normalized using the rlog() function from DESeq2 [32]. KEGG BRITE was used to classify KOs into functional hierarchies: KEGG Orthology categories, subcategories, and pathways were retrieved from the KEGG Pathway database [31]. Transcriptional investment toward different cellular processes was estimated by calculating the proportion of counts belonging to each KEGG subcategory in each sample. Fractions do not add to 100% because KOs can belong to zero or multiple subcategories.

To measure changes in gene expression, we used the Starved condition as the reference point. This is because our experimental cultures began in this condition and then returned to this baseline again at the end of our experiment. The Starved sample was also used as the baseline and time 0 for the KN expression for continuity and to allow comparisons in expression between recently sequestered nuclei and those that have been retained in the host *M. chamaeleon* for up to 2 weeks. Differentially expressed genes were identified using the DESeq2 package [32]. Log2-fold changes (LFCs) were calculated using apeglm [33], as implemented in the lfcShrink() function from DESeq2. KOs with LFC > 0.263 (1.2-fold change) and Benjamini–Hochberg-adjusted *P*-value <.05 were considered to be differentially expressed. Gene set enrichment analysis (GSEA), as implemented in the ClusterProfiler package [33], was used to identify KEGG classifications that show statistically significant differences in gene expression between each treatment and the Starved control. Net enrichment scores were calculated by supplying LFCs and gene sets from KEGG subcategory or KEGG pathway classification to the GSEA() function, along with flags “minGSSize = 5, maxGSSize = 1000, seed = TRUE, pvalueCutoff = 1.” Statistical significance was determined by the false discovery rate (*q*-value <0.05).

## Results

### Retained organelles become increasingly diffuse and distorted during starvation

**Figure 1:**
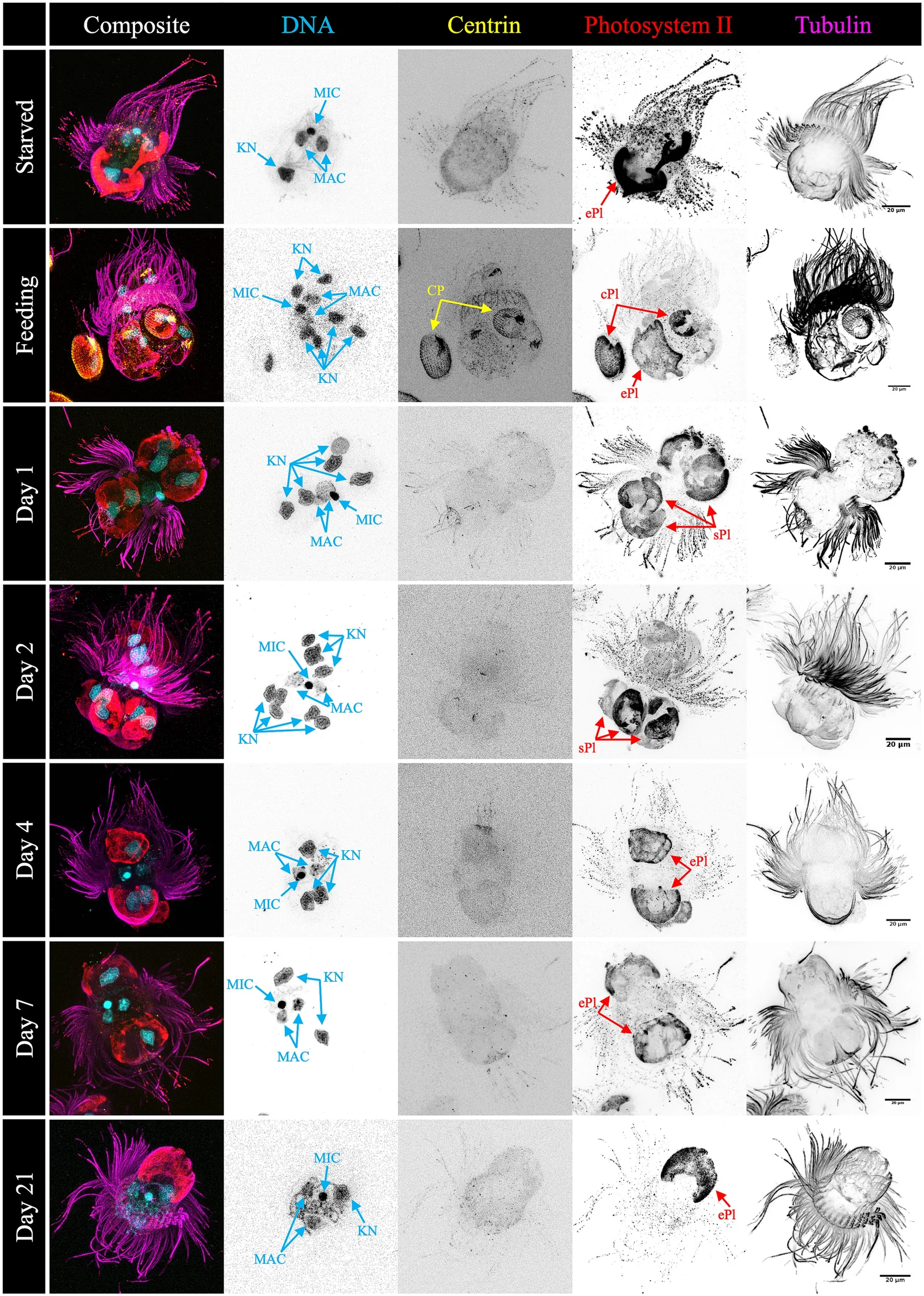
Sequestered organelles expand and become more diffuse and the number of kleptokarya decreases after ingestion. Fluorescent micrographs of *M. chamaeleon* ultrastructure when starved (top) and at 5 h (“Feeding”), Day 1, Day 2, Day 4, Day 7, and Day 21 (bottom) post-pulse feeding. Images were obtained using expansion microscopy techniques. The fluorescent signal from each individual channel is shown alongside the pseudocolored composite image. Nucleic acid was stained with SYBR Green (cyan), and immunohistochemistry was performed to fluorescently label centrin (yellow), the photosystem II D1 protein (red), and alpha and beta tubulin (magenta). A scale bar of 20 μm is displayed in the bottom-right corner of each panel. Ciliate macronuclei (MAC), micronuclei (MIC), and cryptophyte kleptokarya (KN) are annotated in the DNA channel, and the cryptophyte periplast (CP) is noted in the Centrin channel. In the Photosystem II channel, different plastid morphology types are highlighted. The cryptophyte plastid (cPl) appears structurally similar to that of free-living cryptophytes (as seen in the “Feeding” timepoint), sequestered plastids (sPls) are no longer surrounded by the cryptophyte periplast but remain cup-shaped, and the enlarged plastids (ePls) assume a distinct morphology from that of free-living cryptophytes.

Expansion microscopy images (Fig. 1, Fig. S1) confirmed considerable feeding post-starvation, with *M. chamaeleon* transitioning from a single sequestered plastid and cryptophyte nucleus (KN) (Fig. 1, Starved) to containing up to eight newly ingested prey cells (Fig. 1, Day 2; Fig. S1a, mean = 4.5 prey cells for Day 1 and Day 2). These prey cells remained largely intact within the first 24 h, as evidenced by their similar morphology to free-living cryptophyte cells and the presence of the cryptophyte periplast highlighted by centrin-conjugated fluorescent antibodies (Fig. 1). After 1 day, this centrin signal disappeared, suggesting digestion or removal of the cryptophyte’s exterior structure. Throughout the timeline, a single KN remained closely associated with each plastid (Fig. S1b) within a sequestered organelle complex (SOC) most likely bound by a ciliate digestive vacuole [15].

After the Day 2 timepoint, sequestered plastids substantially changed morphology; plastids enlarged and wrapped around the exterior of the cell, adopting inflated or finger-like morphologies in some images taken after the Day 2 timepoint and in all images from the Starved timepoint (e.g., Fig. 1: “Starved”). Stark examples can be seen by comparing the plastid structure of free-living or recently (<24 h) ingested cells (Fig. 1: “Feeding” or “Day 1”), with that of *M. chamaeleon* cells that have not consumed fresh prey material for weeks (Fig. 1: “Starved” or “Day 21”; Fig. 1 “Feeding” Photosystem II annotations). This suggests that plastids become increasingly distorted the longer they are retained in the *M. chamaeleon* cell. In images taken <24 h after feeding, inflated plastids can be seen alongside newly ingested cells, but after 24 h, only enlarged plastids remain.

### Pulse feeding produces transient increases in photosynthetic activity and growth rates

**Figure 2:**
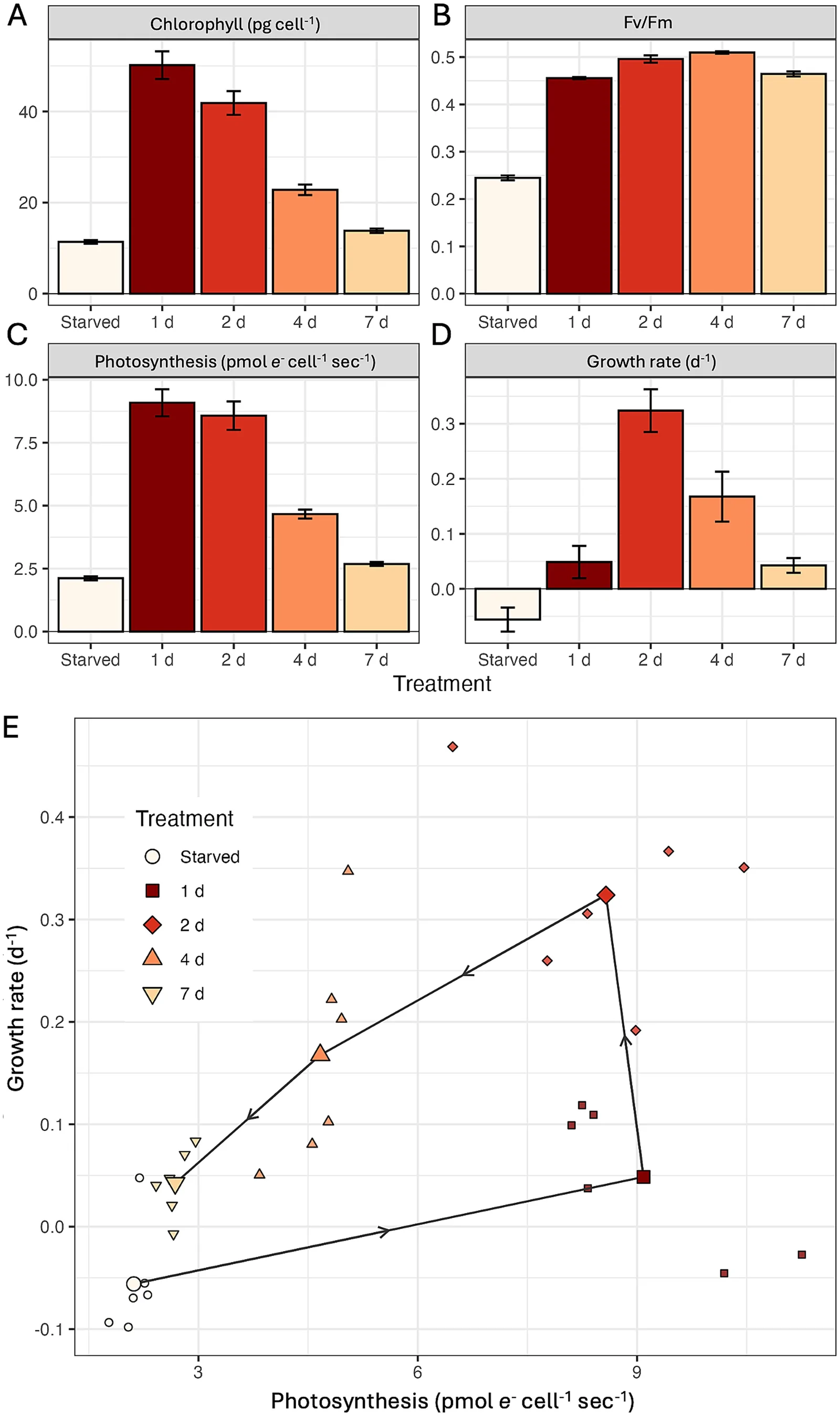
*Mesodinium chamaeleon* experiences transient photosynthetically supported growth pulses after feeding. Physiology measurements for starved cells (unfed for 21 days) and for samples taken 1, 2, 4, and 7 days after pulse feeding with *S. major*. (A) Chlorophyll content, quantum efficiency of photosystem II (Fv/Fm), per-cell photosynthetic electron transport rate, and 3-day-averaged population growth rate at each timepoint. Error bars indicate the standard error of the mean across six replicates. (B) Scatter plot of 3-day-averaged population growth rate versus individual photosynthetic rate. The large shapes indicate the centroid of each timepoint. Centroids are connected in chronological order.

*Mesodinium chamaeleon* retained plastids (11.37 ± 0.41 pg chl cell^−1^) and photosynthetic capacity (0.14 ± 0.01 pmol electrons cell^−1^ s^−1^) after 3 weeks of starvation (Fig. 2A and C, “Starved”), consistent with previous studies [16, 34]. Per-cell chlorophyll content and per-cell photosynthetic rates increased to a maximum 1 day after the feeding event (50.17 ± 3.03 pg chl cell^−1^ and 0.59 ± 0.03 pmol electrons cell^−1^ s^−1^, respectively) and then precipitously fell along the post-feeding timeline (Fig. 2A). The quantum yield of PSII (Fv/Fm), a proxy for photosynthetic energy conversion, peaked at Day 4 post-feeding (0.51 ± 0.002), and remained at a similar magnitude 7 days post-feeding (0.46 ± 0.01) (Fig. 2B). Changes in plastid morphology coincided with the timing of decreased photosynthetic rate.

*Mesodinium chamaeleon* growth rates showed a delayed response to ingestion and retention of photosynthetically active machinery (Fig. 2D). Specifically, growth rates reached a maximum of 0.33 ± 0.04 cells day^−1^ 2 days after feeding. As with the ETR-based photosynthetic rate, growth rates declined at the Day 4 and Day 7 post-feeding timepoints, approaching the Starved growth rate. The number of sequestered KNs and plastids per cell decreased after almost all prey was cleared (Fig. S2) and the highest growth rates were achieved on Day 2 (Figs 1 and 2, Fig. S1), consistent with the loss of sequestered organelle complexes to daughter cells during division.

*Mesodinium chamaeleon* growth rates were positively correlated with ETR-based photosynthetic rates, beginning 2 days post-ingestion (Fig. 2E). These data suggest a sequence of growth limitation phases during the transitions from starved to fed and starved again. First, ingestion and sequestration of new plastids boost photosynthetic rates while growth rates lag; we posit that this lag phase emerges from *M. chamaeleon* upregulating growth machinery (see next section). Second, growth rates are high and correspond with high photosynthetic rates, but the observed lag in growth rate relative to saturated photosynthetic rates indicates that photosynthate is not the sole growth-limiting resource. Finally, as prey organelles are depleted, limited photosynthetic activity leads to attenuation in growth. When exposed to prey after starvation, *M. chamaeleon* is able to quickly ingest, process, and use prey material for photosynthesis, but a single pulse-feeding event is unable to sustain high photosynthetic and growth rates across generations.

### Transcriptional investment into functional KO groups is more variable in the KN than the *M. chamaeleon* macronucleus through the experimental timeline

We investigated the transcriptional responses to prey ingestion and starvation using RNA-Seq. In total, the *M. chamaeleon* transcriptome contained 1694 unique KOs assigned to the *M. chamaeleon* macronuclei (MAC) and 4343 unique KOs assigned to the *S. major* kleptokaryon (KN). As we previously reported, the MAC is missing genes for many metabolic and anabolic pathways, including the pentose phosphate pathway and some amino acid and lipid biosyntheses [28]. Nearly all MAC KOs (1648) were expressed in more than one timepoint, whereas only 60% of KN KOs (2620) were expressed at this threshold. The relatively low KN fraction may be a consequence of a small effective library size, because the KN comprised only 3%–5% of mapped reads (Fig. S3). The fraction of counts assigned to the KN decreased with time since feeding, consistent with a decline in the quality or quantity of genetic material in the kleptokaryon. The first two principal components captured 81% of variance in the MAC data and 80% in the KN, and the biplot showed the greatest similarities between Days 1 and 2 and between Days 4 and 7 (Fig. S4).

**Figure 3:**
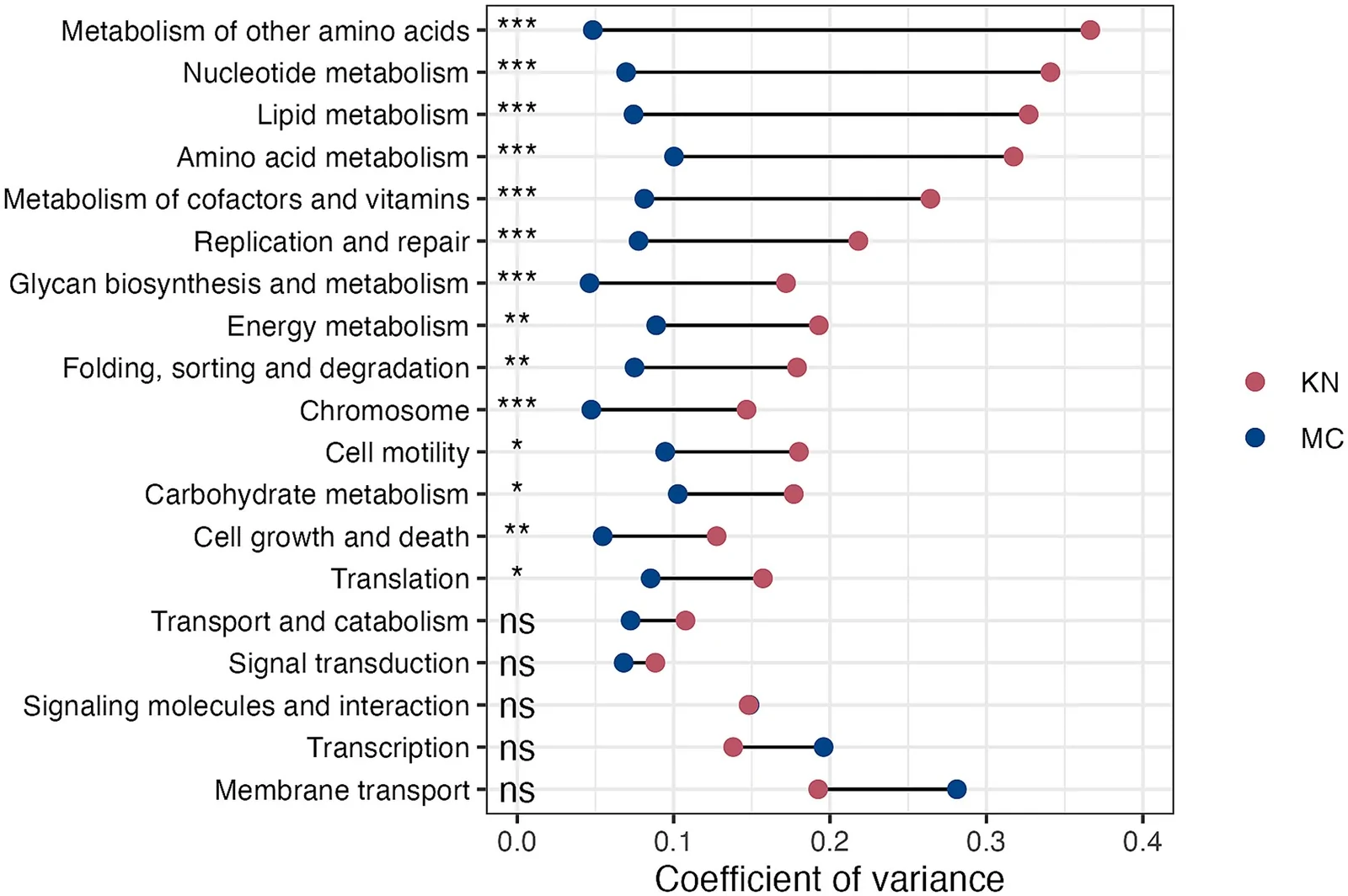
Relative expression of KO functional groups is more variable in the KN than in the MAC. Coefficients of variation for MAC (blue) and KN (red) for each category across all experimental timepoints. Significant differences in coefficient of variation were found by the Feltz and Miller test [35], as implemented by the *cvequality* package in R (^***^, *P* < .001; ^**^, *P* < .01; ^*^, *P* < .05; ns, no significant difference).

We estimated transcriptional investment in different cellular processes by comparing the fraction of length-normalized counts assigned to each KEGG subcategory. Across timepoints, the KN had a significantly greater coefficient of variance in transcriptional investment compared with the MAC in 14 of 19 KEGG subcategories (Feltz and Miller test; Fig. 3; Fig. S5). Comparatively variable KN expression is supported by the greater standard deviation in the log-fold change of gene expression in the KN (0.98) than in *M. chamaeleon* (0.40). Shifts in transcriptional investment over the timeline were positively correlated between the MAC and the KN for the KEGG subcategories transport and catabolism, cell motility, replication and repair, chromosome, and nucleotide metabolism (Fig. S6), and most highly for the nitrogen metabolism and DNA replication pathways (Table S1). In contrast, the subcategories translation, lipid metabolism, and membrane transport, as well as the ribosome and pyruvate metabolism pathways, were each negatively correlated between nuclear complexes. Sequestered KN expression showed marked differences from free-living *S. major* cells (Figs S7 and S8). Only 2 of the 19 most highly expressed functional categories (translation and signaling molecules and interaction) did not show a significant difference in expression between the free-living and sequestered groups.

### MAC and KN genes are most differentially expressed directly after feeding and sequestration

**Figure 4:**
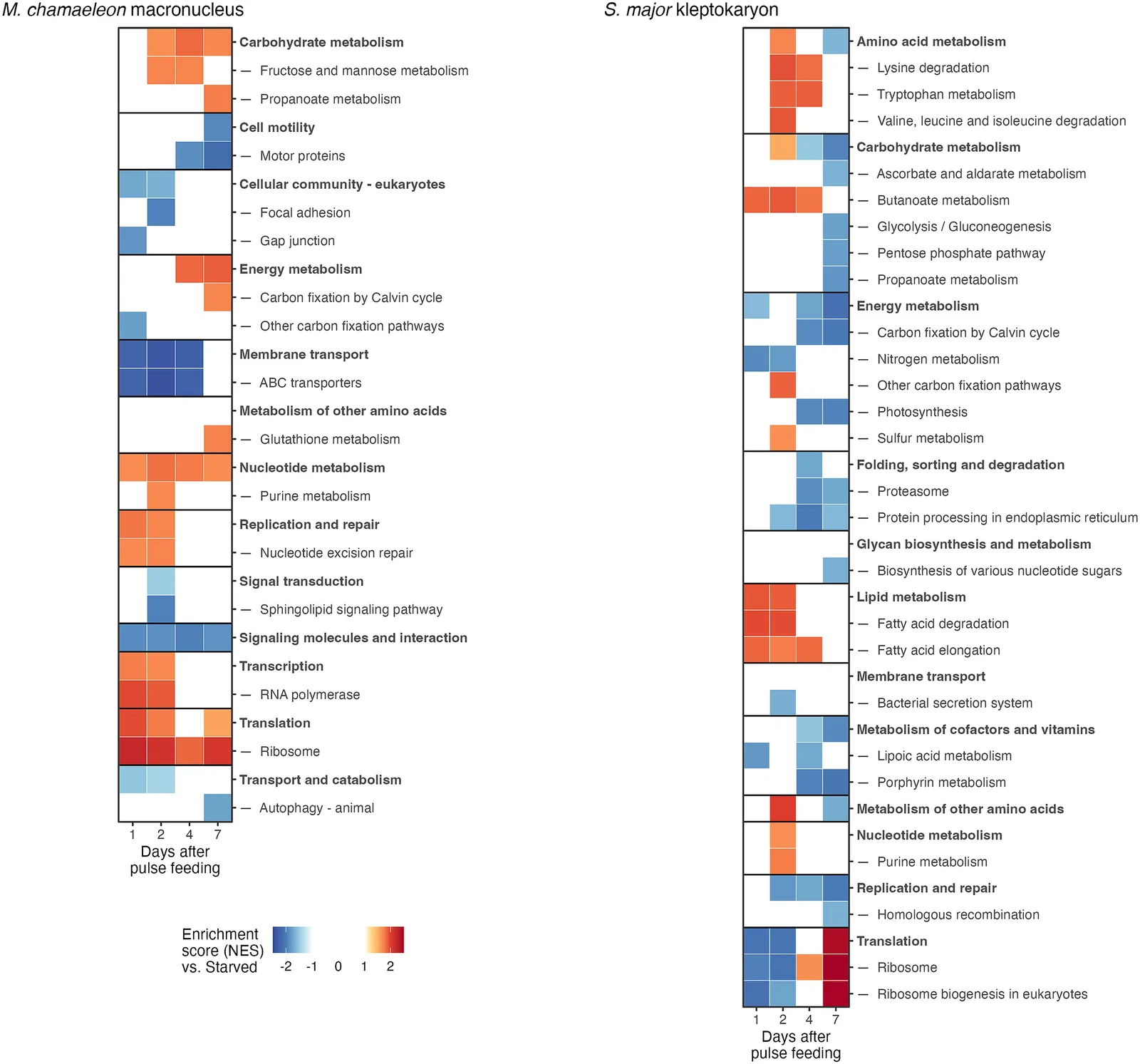
Gene set enrichment analysis (GSEA) results for KEGG subcategories and pathways relative to the Starved condition. Only significantly enriched gene sets (*q*-value <0.05) are colored by their normalized enrichment score (NES).

Compared with the Starved condition baseline, the greatest deviations in gene expression were found in the Day 1 and Day 2 post-feeding timepoints, followed by a decay back toward the Starved condition as time progressed (Fig. S9). Gene Set Enrichment Analysis (GSEA) was used to identify functional categories with statistically significant, concordant differences between each fed condition and the Starved control. In the MAC, metabolism (carbohydrate, energy, nucleotide, xenobiotics degradation, and metabolism) and genetic information processing (transcription, translation, replication, and repair) functional categories were enriched with significantly upregulated genes at various timepoints after feeding (Fig. 4). Conversely, ciliate environmental information processing (membrane transport, signal transduction, signaling molecules, and interaction) and cellular processes (transport and catabolism, cell motility, cell growth, and death) categories were significantly downregulated.

A greater number of subcategories and pathways were enriched with significantly differentially expressed genes in the KN. As opposed to the unidirectional regulation of MAC genes grouped by functional category across the post-feeding timeline, disparate regulation patterns existed between pathways within the same subcategories in the KN; all major categories showed enrichment in both upregulated and downregulated genes, and some pathways showed dramatic swings in enrichment values over the feeding timeline (e.g., ribosome-related pathways) (Fig. 4). The photosynthesis pathway was enriched with downregulated genes at Day 4 and Day 7 post-feeding, coinciding with an increase in nitrate acquisition genes (Fig. 4; Fig. S10).

### Transcription of functional groups reveals more explicit relationships with photosynthetic rate than with growth rate

**Figure 5:**
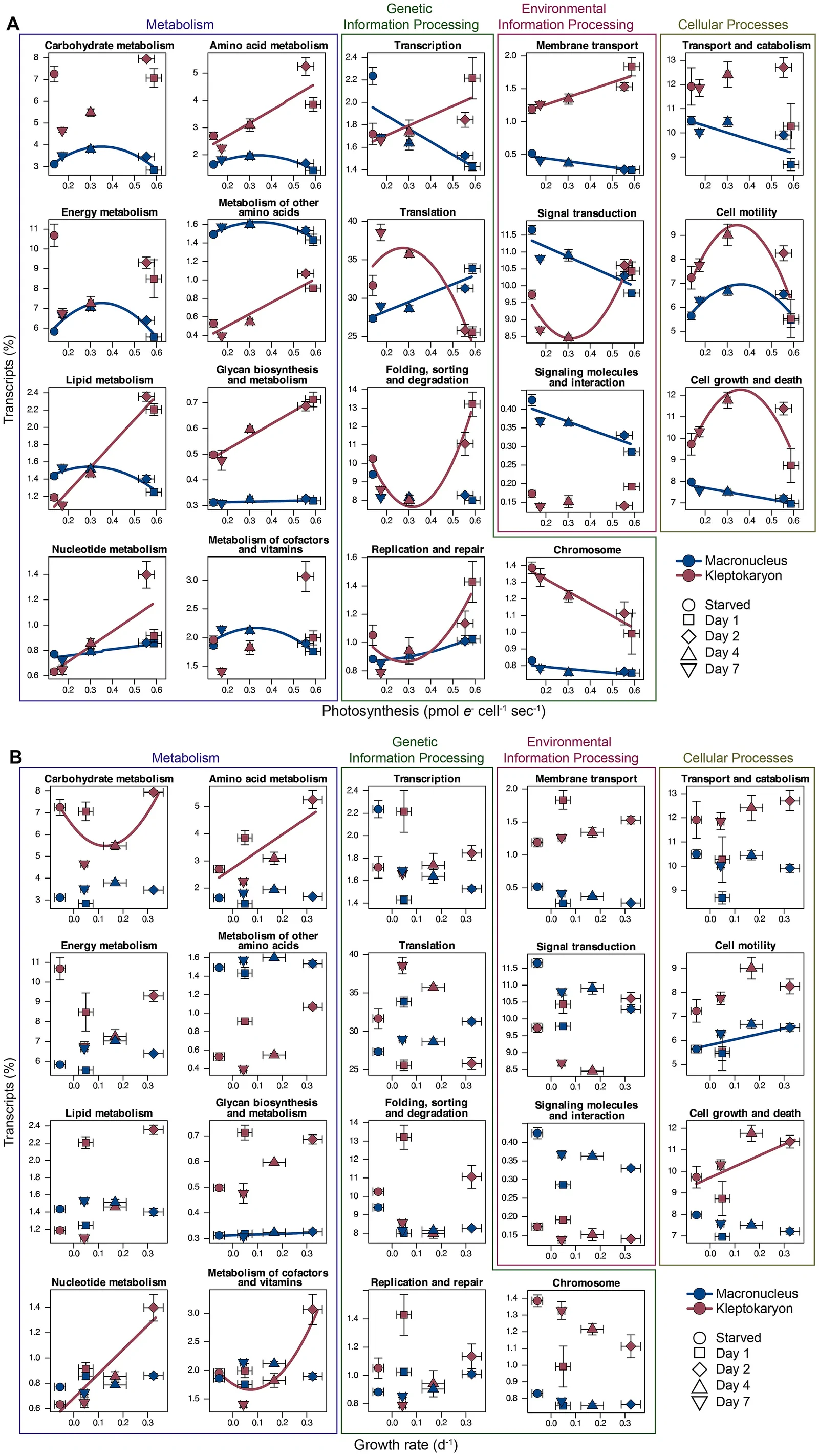
Correlations between the relative expression pattern of genes across the experimental timeline, displayed as a fraction of the reads mapped to the organism’s total library at each timepoint, and *M. chamaeleon* (A) photosynthetic rate (B) growth rate. The best-fits [based on the lowest Akaike Information Criterion (AIC) value for evaluating model quality] of linear and quadratic nonlinear least squares (NLS) models are overlaid when *r*^2^ > 0.5. NLS models were produced by the nls() function in base *r*. These curves do not necessarily indicate significance but rather are used as a tool to visualize general relationships.

The correlations between transcriptional investment and physiology allude to the putative regulation of photosynthesis and *M. chamaeleon* growth by the MAC and KN. Per-subcategory transcriptional investment in the MAC and KN showed a range of responses to the growth rate and photosynthetic rate (Fig. 5). KN lipid, membrane transport, nucleotide metabolism, transcription, and amino acid metabolism displayed positive linear relationships with photosynthesis, and KN amino acid metabolism and nucleotide metabolism showed a positive relationship with *M. chamaeleon* growth rates. The remaining majority of the relationships that surpassed the *R*^2^ > 0.5 threshold were unimodal. The expression of replication and repair, nucleotide metabolism, and cell motility functional categories plotted against photosynthesis followed similar patterns in both *M. chamaeleon* and the KN. At the pathway level, similar patterns in expression in relation to photosynthesis were evident in nitrogen metabolism and purine metabolism (Figs S11 and S12). Generally, the relationships between transcription and growth rate produced fewer well-fitting models and showed few consistencies between the MAC and KN at both the subcategory and pathway levels.

## Discussion

In this study, we evaluated the morphological, physiological, and transcriptional changes that accompany the acquisition, sequestration, degradation, and subsequent loss of functional prey organelles in *M. chamaeleon*. Prey capture appears to produce a cycle of growth initialization followed by attenuation as stolen organelles are integrated and then lost or diluted over time. The high variability in kleptokaryon gene expression (compared to *M. chamaeleon* macronuclear genes; Fig. 3), combined with the changes in cryptophyte nuclear expression between free-living and sequestered cells (Fig. S6) and substantially lower KN contributions to the global ciliate transcriptome relative to *M. rubrum* [13, 36], may be evidence of disrupted pathway regulation in the KN that underlies functional organelle degradation over time. The instability of prey nuclei is considered the bottleneck to functional plastid longevity in *M. rubrum* [1, 9, 13], as well as some kleptoplastidic dinoflagellates [37, 38]. The decline in growth that corresponds with a reduction in the number and functionality of sequestered organelles supports our hypothesis that high prey turnover in *M. chamaeleon* is necessary due to the loss of plastids through cell division and rapid degradation of KN function. Even though sequestered plastids persist in *M. chamaeleon* populations for weeks and retain photosynthetic efficiencies comparable to those of recently ingested plastids, the decline in chlorophyll, per-cell photosynthetic rate, and transcription of plastid-supporting KN metabolic pathways inhibits maximum photosynthesis-supported growth across generations.

Temporal mismatches in photosynthetic rate and growth revealed two phases of growth limitation: recently fed cells are limited by endogenous cellular machinery, whereas cells that have not ingested fresh prey material for >4 days, or daughter cells that receive smaller fractions of plastid machinery, are limited by photosynthetic rate. At the onset of feeding (Day 1 and Day 2 post-feeding timepoints), *M. chamaeleon* growth rates showed an unexpected decoupling from per-cell photosynthetic rate. During the same period, the ciliate also significantly upregulated genes involved in ribosome synthesis and transcription, suggesting that it is performing cellular remodeling. The observed delay in growth after prey ingestion may be a result of the time required for starved cells to build and prepare the cellular machinery required to incorporate and utilize the influx of photosynthate or prey-derived organic carbon, amino acids, nucleotides, and compounds that may not be endogenously synthesized [28]. Growth rate is dependent on ribosomal activity [39], and synthesis of ribosomes and proteins has been described to produce a “lag phase” in the transition between low-growth and exponential growth conditions in prokaryotes [40] and yeast [41].

After *M. chamaeleon* reached maximum growth rates 2 days post-feeding, growth declined with photosynthetic rate, suggesting a secondary phase of growth limitation at the population level by photosynthesis. Declining per-cell photosynthetic rates likely arise from dilution of photosynthetic machinery through division and degradation of plastid functionality, but may be compounded by nutrient limitation: in the KN, the latter two timepoints show significant downregulation of carbon fixation, photosynthesis, glycolysis/gluconeogenesis, and pentose phosphate pathways (Fig. 4; within KN expression under carbohydrate metabolism) that coincide with upregulation of nitrogen assimilation pathways (Fig. S8) characteristic of nitrogen limitation in phytoplankton [42]. Without complete nitrate assimilation pathways [28], it is unclear whether *M. chamaeleon* can supply nitrogen to the plastid, which may limit persistent photosynthetic function relative to *M. rubrum* whose ability to uptake inorganic nitrate supports high rates of photosynthesis in bloom-forming conditions [43]. Additionally, the downregulation of porphyrin synthesis by *S. major* when sequestered compared to free-living may be a result of nitrogen inaccessibility that inhibits photosynthesis [44]. The expression of photosynthesis-related genes did not scale linearly with photosynthetic rate (Fig. S11) and ABC transporters are inversely related to the photosynthetic rate (Fig. S12). ABC transporters are involved in intracellular trafficking of nitrate, among other processes, in photosynthetic organisms [45], implying that although transcription did not limit photosynthesis, low nitrogen availability may have played a role. Fatty acid elongation also increased with photosynthetic rate (Fig. S11) and phytoplankton are known to synthesize nutrient-poor fatty acid molecules to store organic carbon downstream of CO_2_ fixation when nutrients are scarce [46]. These trends do not provide direct empirical evidence of nutrient fluxes, but they are a suggestion that nitrogen acquisition and supply to the stolen plastid may be a factor in photosynthetic performance.

The morphological changes associated with prey ingestion provide context for the emergent physiology and molecular dynamics. Prey cells are structurally modified within hours of ingestion in *M. chamaeleon*—first the cryptophyte periplast and flagella are degraded within 24 h, and then plastid morphology changes. Studies employing transmission electron microscopy in *Mesodinium* support this chronology; In *M. chamaeleon*, the cryptophyte periplast, trichocysts, flagella, Golgi apparatus, and other organelles are lost in mature digestive vacuoles in which mainly plastids and KNs remain [15], and in *M. rubrum*, the cryptophyte periplasts and plasma membranes are destroyed within 12 h of ingestion [47].

SOCs appear to remain intact because sequestered plastids were always paired with a KN in a 1:1 ratio within the cell (Fig. S1, panel B), consistent with electron microscopy observations of the species [15]. Cells were always observed containing at least one prey nucleus and plastid. During starvation, *M. chamaeleon* plastid and KN content asymptotically declined to one plastid-KN pair per ciliate cell (Fig. S1, panel A). This loss may be a result of digestion, removal, or dilution through division because both daughter cells presumably receive an SOC. Along with the apparent selective removal of plastids (Fig. 1), this implies that digestion or extraction of plastids only occur when more than one SOC is available.

Plastid enlargement is a common feature of kleptoplasty [48]. It has been previously observed in *M. chamaeleon* (associated with large amounts of storage material in the form of starch granules) [15], *M. rubrum* [47, 49], and kleptoplastidic dinoflagellates. The dinoflagellates *Nusuttodinium latum, N. myriopyrenoides, N. aeruginosum*, and *Hatena arenicola*, in which plastid enlargement during kleptoplasty has been reported [50–53], also retain the KN. Additionally, in the Ross Sea Dinoflagellate, plastid size and photosynthetic function closely track the enlargement and contraction of the accompanying KN over time [5]. These studies implicate the stolen nucleus as a driver of structural modification of ingested plastids [48]. A clear exception to these patterns may be found in *Dinophysis* spp., which restructure their cryptophyte kleptoplastids from *M. rubrum* into a stellate plastid morphology, despite lacking the cryptophyte nucleus [54]. Whether changes in plastid morphology are an active mechanism to bolster organelle performance or merely a consequence of dysfunction or exposure to a foreign environment is unknown; however, this is consistent with the notion that functional prey nuclei are the bottleneck to persistent photosynthetic function in kleptoplastidic hosts that do not contain the full suite of genes necessary to operate prey organelles independently [1, 9, 36, 48, 55]. The precise relationship between plastid morphology and cell-specific photosynthetic performance in *Mesodinium* also requires further study.

Although acquiring photosynthetic pathways for organic carbon assimilation are among the overt theoretical benefits of kleptoplasty, *M. chamaeleon* also lacks the ability to synthesize some essential amino acids, fatty acids, and nucleotides that can be generated by the prey [28, 49]. Positive linear relationships between KN relative investment in transcribing genes related to amino acid and nucleotide metabolism and *M. chamaeleon* growth rates may point to additional benefits of prey ingestion. Depending on the mechanism of acquiring these compounds (e.g., passive flux through the SOC membrane or digestion of the SOC), *M. chamaeleon* may face a tradeoff between retaining and digesting the organelle complex that is constrained by its ecophysiology. Future empirical studies quantifying resource fluxes from ingested prey and organelle complexes could elucidate the tradeoffs between acquiring organic carbon by harboring kleptoplastids vs. obtaining more essential compounds from digestion. Comparisons across the *Mesodinium* genus may also shed light on whether rapid turnover of prey organelles is a result of the imperfect organelle retention system or a mechanism to achieve high growth rates in well-fed conditions that reflect observed ecological settings [15, 16, 18].

## Conclusion

This pulse feeding to starvation timeline highlights *M. chamaeleon's* cellular rewiring upon consuming prey followed by reductions in the number and functionality of sequestered organelles over time that results in a temporal mismatch between photophysiology and *M. chamaeleon* growth rates. Understanding the molecular mechanisms and resulting physiological and morphological changes accompanying sequestration of prey organelles in kleptoplastidic species may provide clues to the physiological ecology and morphology of photoendosymbiotic intermediates. However, we emphasize the importance of conceptualizing kleptoplasty in *M. chamaeleon* as a potentially stable ecological strategy that acknowledges benefits ranging from transient photosynthetic capacity to the acquisition of necessary biosynthesis pathways and offloading energetically expensive endogenous regulatory networks. We recommend that future research addresses gaps in our understanding of digestion dynamics through live cell imaging, identify exchanged metabolites through metabolomics and isotope tracing, and determine the localization of prey-derived compounds in the host through spatial proteomics or spatially resolved mass spectrometry approaches such as NanoSIMS.

## Supplementary Material

Supplementary_material_wrag204

## Data Availability

Physiological data and all R scripts used in this paper are available in the Supplemental Dataset: doi:10.5281/zenodo.21112941. RNASeq data for *M. chamaeleon* NRMC1501 are available in NCBI under the BioProjects:PRJNA560220. RNASeq data generated in this study for *M. chamaeleon* NRMC1802 and *Storeatula major* are available through the JGI Genome Portal https://genome.jgi.doe.gov/portal/ under the proposal ID 503035. Transcriptome assemblies, predicted coding sequences, protein annotations, and other associated data are available in Dryad: doi:10.5061/dryad.1jwstqk4x (reviewer link: http://datadryad.org/stash/share/s3Uh8hHzyr0bUMOftrqKg2miXVxD6l4VVaQp0YtWI5E).
