# Supplementary material for: Integration and regulation of stolen organelles in a marine protist
Source: ISME J. 2026 Aug 4;20(1):wrag204. doi: 10.1093/ismejo/wrag204 (PMC13592161; doi:10.1093/ismejo/wrag204)
Supplement: Supplementary file 1 [file supplementary_material_wrag204.zip › Supplementary_Information_wrag204.docx]

# Supplementary Information

**Figure S1**. (A) Mean (± se) number of KN per *M. chamaeleon* cell from starvation to 1, 2, 4, 7, and 21 days post-feeding. (B) Scatterplot of number of KN and number of distinguishable plastids per *M. chamaeleon* cell with dotted 1:1 line and solid linear model fit. (C) Scatterplot of chlorophyll per cell against the number of plastids within a *M. chamaeleon* cell.

**Figure S2.** Mean *M. chamaeleon* cell density (top), growth rate (middle), and ratio of *S. major* to *M. chamaeleon* cells (bottom) with standard error during the pre-experiment acclimation phase and during experiment sampling. Experimental timepoints are denoted with a vertical dashed line.

**Figure S3**. The fraction of reads mapped to the *S. major* kleptokaryon (KN) for starved cells (unfed for 21 d) and for samples taken 1, 2, 4, and 7 d after pulse feeding with *S. major*. The fraction was calculated as the number of reads mapped to KN KOs divided by the number of reads mapped to either KN or MAC KOs; unmapped reads are not included here.

**Figure S4**. Biplots of the first and second principal components (PCs) derived from the macronucleus (top) and kleptokaryon (bottom) KO-mapped counts.

**Figure S5**. Mean +/- se of fraction of reads belonging to the ten KEGG subcategories with the highest total expression. The Y axis is scaled log2. The Starved timepoint is included again at the end of the timeseries (open circle).

**Figure S6.** Coordination of MAC and KN gene expression varies by functional group. Mean +/- se of percent of reads belonging to *Mesodinium chamaeleon* (blue) and the kleptokaryon of cryptophyte origin (red) in select KEGG subcategories. The starved timepoint is included again at the end of the timeseries (open circle) and functional categories are organized in decreasing fraction of total reads (left to right, top to bottom). The Pearson correlation coefficient (PCC) between the macronucleus and kleptokaryon fractions is shown if p < 0.05.

**Figure S7.** Biplots of the first and second principal components (PCs) derived from the macronuclear and kleptokaryonic KO-mapped counts.

**Figure S8**. Fractions of reads mapped to select KEGG subcategories for free living *S. major* prey and the sequestered kleptokaryon. Asterisks indicate a significant difference in means (p-values from student’s t test; * < 0.05, ** < 0.01, *** < 0.001, **** < 0.0001, ns = no significance).

**Figure S9**. Proportions of differentially expressed genes (red = upregulated, grey = no differential expression, blue = downregulated) compared to a Starved control decreases with duration of prey sequestration.

**Figure S10**. Expression of kleptokaryon nitrogen acquisition genes during the time series. Counts were normalized using the rlog() function from DESeq2.

**Figure S11**. Correlations between the relative expression pattern of genes across the experimental timeline, displayed as a fraction of the reads mapped to the organism’s total library at each timepoint, and *M. chamaeleon* photosynthetic rate. The best-fits (based on the lowest Akaike Information Criterion (AIC) value for evaluating model quality) of linear and quadratic NLS models are overlaid when *R*^2^ > 0.5. NLS models were produced by the nls() function in base r. These curves do not necessarily indicate significance, but rather are used as a tool to visualize general relationships.

**Figure S12**. Correlations between the relative expression pattern of genes across the experimental timeline, displayed as a fraction of the reads mapped to the organism’s total library at each timepoint, and *M. chamaeleon* growth rate. The best-fits (based on the lowest Akaike Information Criterion (AIC) value for evaluating model quality) of linear and quadratic NLS models are overlaid when *R*^2^ > 0.5. NLS models were produced by the nls() function in base r. These curves do not necessarily indicate significance, but rather are used as a tool to visualize general relationships.

**Table S1.** Correlations (Pearson) in expression of pathways between the MAC and the KN.

| **Pathway** | **Correlation** | **p value** | **Pathway-Associated KOs expressed in MC** | **Pathway-associated KOs expressed in KN** |
| --- | --- | --- | --- | --- |
| Calcium signaling pathway [PATH:ko04020] | -0.923 | 2.51E-06 | 20 | 49 |
| Cellular senescence [PATH:ko04218] | -0.920 | 3.12E-06 | 27 | 36 |
| Glycerolipid metabolism [PATH:ko00561] | -0.844 | 1.49E-04 | 13 | 25 |
| Phosphatidylinositol signaling system [PATH:ko04070] | -0.830 | 2.39E-04 | 16 | 30 |
| Necroptosis [PATH:ko04217] | -0.807 | 4.90E-04 | 12 | 28 |
| MAPK signaling pathway - plant [PATH:ko04016] | -0.794 | 6.90E-04 | 8 | 17 |
| Sulfur relay system [PATH:ko04122] | -0.734 | 0.003 | 5 | 6 |
| Glycerophospholipid metabolism [PATH:ko00564] | -0.713 | 0.004 | 17 | 38 |
| Pyruvate metabolism [PATH:ko00620] | -0.709 | 0.005 | 13 | 36 |
| Peroxisome [PATH:ko04146] | -0.681 | 0.007 | 14 | 32 |
| cGMP-PKG signaling pathway [PATH:ko04022] | -0.677 | 0.008 | 16 | 36 |
| ErbB signaling pathway [PATH:ko04012] | -0.665 | 0.009 | 12 | 13 |
| Ribosome [PATH:ko03010] | -0.658 | 0.011 | 84 | 109 |
| Nucleocytoplasmic transport [PATH:ko03013] | -0.639 | 0.014 | 19 | 52 |
| mTOR signaling pathway [PATH:ko04150] | -0.625 | 0.017 | 24 | 47 |
| Other carbon fixation pathways [PATH:ko00720] | -0.624 | 0.017 | 6 | 14 |
| Fructose and mannose metabolism [PATH:ko00051] | -0.613 | 0.020 | 8 | 19 |
| Hippo signaling pathway - fly [PATH:ko04391] | -0.611 | 0.020 | 9 | 15 |
| mRNA surveillance pathway [PATH:ko03015] | -0.610 | 0.021 | 20 | 48 |
| Methane metabolism [PATH:ko00680] | -0.608 | 0.021 | 9 | 18 |
| Protein export [PATH:ko03060] | -0.561 | 0.037 | 11 | 23 |
| Glutathione metabolism [PATH:ko00480] | -0.557 | 0.038 | 14 | 20 |
| Signaling pathways regulating pluripotency of stem cells [PATH:ko04550] | -0.542 | 0.045 | 6 | 10 |
| Lysosome [PATH:ko04142] | -0.539 | 0.046 | 34 | 48 |
| Carbon fixation by Calvin cycle [PATH:ko00710] | -0.490 | 0.076 | 10 | 22 |
| Citrate cycle (TCA cycle) [PATH:ko00020] | -0.485 | 0.079 | 17 | 18 |
| Cysteine and methionine metabolism [PATH:ko00270] | -0.449 | 0.107 | 8 | 37 |
| Pentose phosphate pathway [PATH:ko00030] | -0.441 | 0.114 | 5 | 19 |
| ABC transporters [PATH:ko02010] | -0.424 | 0.130 | 15 | 30 |
| Sphingolipid signaling pathway [PATH:ko04071] | -0.423 | 0.132 | 19 | 26 |
| Rap1 signaling pathway [PATH:ko04015] | -0.410 | 0.145 | 10 | 24 |
| Cytoskeleton in muscle cells [PATH:ko04820] | -0.389 | 0.169 | 10 | 38 |
| Endocytosis [PATH:ko04144] | -0.367 | 0.196 | 29 | 72 |
| Starch and sucrose metabolism [PATH:ko00500] | -0.366 | 0.198 | 9 | 22 |
| Hippo signaling pathway [PATH:ko04390] | -0.364 | 0.201 | 10 | 23 |
| Glyoxylate and dicarboxylate metabolism [PATH:ko00630] | -0.357 | 0.211 | 9 | 26 |
| Viral life cycle - HIV-1 [PATH:ko03250] | -0.338 | 0.237 | 12 | 19 |
| Ribosome biogenesis in eukaryotes [PATH:ko03008] | -0.334 | 0.243 | 37 | 57 |
| Fatty acid degradation [PATH:ko00071] | -0.309 | 0.282 | 5 | 16 |
| TNF signaling pathway [PATH:ko04668] | -0.308 | 0.284 | 10 | 10 |
| Hormone signaling [PATH:ko04081] | -0.277 | 0.337 | 5 | 11 |
| Porphyrin metabolism [PATH:ko00860] | -0.276 | 0.340 | 6 | 27 |
| Glycolysis / Gluconeogenesis [PATH:ko00010] | -0.267 | 0.356 | 17 | 32 |
| Nicotinate and nicotinamide metabolism [PATH:ko00760] | -0.260 | 0.370 | 8 | 17 |
| Two-component system [PATH:ko02020] | -0.248 | 0.392 | 6 | 19 |
| Inositol phosphate metabolism [PATH:ko00562] | -0.239 | 0.411 | 14 | 27 |
| Homologous recombination [PATH:ko03440] | -0.203 | 0.487 | 8 | 20 |
| Ras signaling pathway [PATH:ko04014] | -0.174 | 0.552 | 15 | 29 |
| MAPK signaling pathway [PATH:ko04010] | -0.169 | 0.563 | 22 | 48 |
| Arginine and proline metabolism [PATH:ko00330] | -0.140 | 0.632 | 8 | 21 |
| Alanine, aspartate and glutamate metabolism [PATH:ko00250] | -0.130 | 0.657 | 6 | 28 |
| VEGF signaling pathway [PATH:ko04370] | -0.130 | 0.658 | 9 | 9 |
| MAPK signaling pathway - fly [PATH:ko04013] | -0.122 | 0.679 | 10 | 23 |
| Mitophagy - yeast [PATH:ko04139] | -0.102 | 0.729 | 10 | 15 |
| Regulation of actin cytoskeleton [PATH:ko04810] | -0.096 | 0.743 | 14 | 31 |
| cAMP signaling pathway [PATH:ko04024] | -0.095 | 0.747 | 23 | 40 |
| Tyrosine metabolism [PATH:ko00350] | -0.074 | 0.800 | 6 | 13 |
| Glycine, serine and threonine metabolism [PATH:ko00260] | -0.064 | 0.829 | 6 | 30 |
| Apoptosis - fly [PATH:ko04214] | -0.056 | 0.849 | 5 | 18 |
| Adherens junction [PATH:ko04520] | -0.029 | 0.922 | 5 | 16 |
| Terpenoid backbone biosynthesis [PATH:ko00900] | -0.009 | 0.976 | 7 | 30 |
| p53 signaling pathway [PATH:ko04115] | 0.008 | 0.978 | 12 | 13 |
| Non-homologous end-joining [PATH:ko03450] | 0.023 | 0.937 | 6 | 6 |
| PI3K-Akt signaling pathway [PATH:ko04151] | 0.048 | 0.871 | 29 | 46 |
| Glycosylphosphatidylinositol (GPI)-anchor biosynthesis [PATH:ko00563] | 0.075 | 0.800 | 5 | 16 |
| Base excision repair [PATH:ko03410] | 0.113 | 0.701 | 16 | 28 |
| MAPK signaling pathway - yeast [PATH:ko04011] | 0.114 | 0.697 | 11 | 17 |
| Aminoacyl-tRNA biosynthesis [PATH:ko00970] | 0.119 | 0.685 | 23 | 25 |
| FoxO signaling pathway [PATH:ko04068] | 0.120 | 0.684 | 20 | 28 |
| Spliceosome [PATH:ko03040] | 0.123 | 0.676 | 12 | 90 |
| Propanoate metabolism [PATH:ko00640] | 0.130 | 0.658 | 10 | 24 |
| AMPK signaling pathway [PATH:ko04152] | 0.160 | 0.586 | 16 | 35 |
| TGF-beta signaling pathway [PATH:ko04350] | 0.164 | 0.575 | 9 | 13 |
| Folate biosynthesis [PATH:ko00790] | 0.170 | 0.562 | 6 | 15 |
| Meiosis - yeast [PATH:ko04113] | 0.174 | 0.553 | 22 | 35 |
| Sphingolipid metabolism [PATH:ko00600] | 0.183 | 0.531 | 8 | 15 |
| Hedgehog signaling pathway - fly [PATH:ko04341] | 0.217 | 0.456 | 10 | 9 |
| Efferocytosis [PATH:ko04148] | 0.228 | 0.433 | 14 | 31 |
| Cell cycle - yeast [PATH:ko04111] | 0.233 | 0.422 | 31 | 52 |
| Phospholipase D signaling pathway [PATH:ko04072] | 0.235 | 0.418 | 9 | 21 |
| Lysine degradation [PATH:ko00310] | 0.246 | 0.396 | 7 | 27 |
| JAK-STAT signaling pathway [PATH:ko04630] | 0.266 | 0.358 | 6 | 6 |
| ATP-dependent chromatin remodeling [PATH:ko03082] | 0.276 | 0.340 | 15 | 37 |
| RNA degradation [PATH:ko03018] | 0.285 | 0.324 | 20 | 47 |
| HIF-1 signaling pathway [PATH:ko04066] | 0.296 | 0.305 | 19 | 26 |
| Mismatch repair [PATH:ko03430] | 0.313 | 0.276 | 15 | 20 |
| Autophagy - animal [PATH:ko04140] | 0.319 | 0.266 | 32 | 66 |
| Cell cycle [PATH:ko04110] | 0.325 | 0.257 | 38 | 65 |
| Wnt signaling pathway [PATH:ko04310] | 0.329 | 0.251 | 18 | 28 |
| Amino sugar and nucleotide sugar metabolism [PATH:ko00520] | 0.333 | 0.244 | 9 | 22 |
| Plant hormone signal transduction [PATH:ko04075] | 0.344 | 0.228 | 5 | 12 |
| Lipoic acid metabolism [PATH:ko00785] | 0.363 | 0.202 | 7 | 15 |
| Fanconi anemia pathway [PATH:ko03460] | 0.368 | 0.196 | 8 | 22 |
| Polycomb repressive complex [PATH:ko03083] | 0.371 | 0.191 | 10 | 22 |
| Hedgehog signaling pathway [PATH:ko04340] | 0.390 | 0.169 | 9 | 12 |
| Focal adhesion [PATH:ko04510] | 0.395 | 0.162 | 19 | 31 |
| Oocyte meiosis [PATH:ko04114] | 0.405 | 0.151 | 28 | 40 |
| Oxidative phosphorylation [PATH:ko00190] | 0.564 | 0.036 | 34 | 47 |
| Autophagy - yeast [PATH:ko04138] | 0.569 | 0.034 | 19 | 47 |
| Apelin signaling pathway [PATH:ko04371] | 0.591 | 0.026 | 16 | 31 |
| One carbon pool by folate [PATH:ko00670] | 0.611 | 0.020 | 6 | 20 |
| Apoptosis [PATH:ko04210] | 0.627 | 0.016 | 19 | 32 |
| Ubiquitin mediated proteolysis [PATH:ko04120] | 0.631 | 0.016 | 32 | 67 |
| Purine metabolism [PATH:ko00230] | 0.652 | 0.011 | 23 | 74 |
| Mitophagy - animal [PATH:ko04137] | 0.702 | 0.005 | 18 | 32 |
| Nucleotide excision repair [PATH:ko03420] | 0.735 | 0.003 | 23 | 44 |
| Protein processing in endoplasmic reticulum [PATH:ko04141] | 0.761 | 0.002 | 36 | 77 |
| Proteasome [PATH:ko03050] | 0.768 | 0.001 | 30 | 37 |
| RNA polymerase [PATH:ko03020] | 0.805 | 5.13E-04 | 16 | 23 |
| Pyrimidine metabolism [PATH:ko00240] | 0.814 | 3.97E-04 | 15 | 29 |
| Phagosome [PATH:ko04145] | 0.853 | 1.07E-04 | 21 | 38 |
| Drug metabolism - other enzymes [PATH:ko00983] | 0.873 | 4.67E-05 | 9 | 14 |
| DNA replication [PATH:ko03030] | 0.888 | 2.17E-05 | 21 | 27 |
| Tight junction [PATH:ko04530] | 0.901 | 1.08E-05 | 12 | 36 |
| Motor proteins [PATH:ko04814] | 0.916 | 4.24E-06 | 43 | 58 |
| Nitrogen metabolism [PATH:ko00910] | 0.925 | 2.22E-06 | 6 | 11 |
| Gap junction [PATH:ko04540] | 0.938 | 7.05E-07 | 12 | 22 |
